# Short-term outcomes of health-related quality of life in patients with locally recurrent rectal cancer: multicentre, international, cross-sectional cohort study

**DOI:** 10.1093/bjsopen/zrac168

**Published:** 2023-02-14

**Authors:** Deena P Harji, Niamh McKigney, Cherry Koh, Michael J Solomon, Ben Griffiths, Martyn Evans, Alexander Heriot, Peter M Sagar, Galina Velikova, Julia M Brown

**Affiliations:** Clinical Trials Research Unit, Leeds Institute of Clinical Trials Research, University of Leeds, Leeds, UK; Department of Colorectal Surgery, Manchester University NHS Foundation Trust, Manchester, UK; Clinical Trials Research Unit, Leeds Institute of Clinical Trials Research, University of Leeds, Leeds, UK; Surgical Outcomes Research Centre (SOuRCe), Royal Prince Alfred Hospital, Sydney, NSW, Australia; Faculty of Medicine and Health, Central Clinical School, The University of Sydney, Sydney, NSW, Australia; Royal Prince Alfred Hospital, RPA Institute of Academic Surgery, Sydney, NSW, Australia; Department of Colorectal Surgery, Royal Prince Alfred Hospital, Sydney, NSW, Australia; Surgical Outcomes Research Centre (SOuRCe), Royal Prince Alfred Hospital, Sydney, NSW, Australia; Faculty of Medicine and Health, Central Clinical School, The University of Sydney, Sydney, NSW, Australia; Royal Prince Alfred Hospital, RPA Institute of Academic Surgery, Sydney, NSW, Australia; Department of Colorectal Surgery, Royal Prince Alfred Hospital, Sydney, NSW, Australia; Department of Colorectal Surgery, Manchester University NHS Foundation Trust, Manchester, UK; Department of Colorectal Surgery, Heol Maes Eglwys, Morriston, Swansea, UK; Sir Peter MacCallum Department of Oncology, University of Melbourne, Melbourne, Victoria, Australia; The John Goligher Department of Colorectal Surgery, St James’s University Hospital, Leeds, UK; Leeds Institute of Medical Research, University of Leeds, Leeds, UK; St James’s Institute of Oncology, St James’s University Hospital, Leeds, UK; Clinical Trials Research Unit, Leeds Institute of Clinical Trials Research, University of Leeds, Leeds, UK

## Abstract

**Background:**

Overall survival rates for locally recurrent rectal cancer (LRRC) continue to improve but the evidence concerning health-related quality of life (HrQoL) remains limited. The aim of this study was to describe the short-term HrQoL differences between patients undergoing surgical and palliative treatments for LRRC.

**Methods:**

An international, cross-sectional, observational study was undertaken at five centres across the UK and Australia. HrQoL in LRRC patients was assessed using the European Organisation for Research and Treatment of Cancer (EORTC) QLQ-CR29 and functional assessment of cancer therapy – colorectal (FACT-C) questionnaires and subgroups (curative *versus* palliative) were compared. Secondary analyses included the comparison of HrQoL according to the margin status, location of disease and type of treatment. Scores were interpreted using minimal clinically important differences (MCID) and Cohen effect size (ES).

**Results:**

Out of 350 eligible patients, a total of 95 patients participated, 74.0 (78.0 per cent) treated with curative intent and 21.0 (22.0 per cent) with palliative intent. Median time between LRRC diagnosis and HrQoL assessments was 4 months. Higher overall FACT-C scores denoting better HrQoL were observed in patients undergoing curative treatment, demonstrating a MCID with a mean difference of 18.5 (*P* < 0.001) and an ES of 0.6. Patients undergoing surgery had higher scores denoting a higher burden of symptoms for the EORTC CR29 domains of urinary frequency (*P* < 0.001, ES 0.3) and frequency of defaecation (*P* < 0.001, ES 0.4). Higher overall FACT-C scores were observed in patients who underwent an R0 resection *versus* an R1 resection (*P* = 0.051, ES 0.6). EORTC CR29 scores identified worse body image in patients with posterior/central disease (*P* = 0.021). Patients undergoing palliative chemoradiation reported worse HrQoL scores with a higher symptom burden on the frequency of defaecation scale compared with palliative chemotherapy (*P* = 0.041).

**Conclusion:**

Several differences in short-term HrQoL outcomes between patients undergoing curative and palliative treatment for LRRC were documented. Patients undergoing curative surgery reported better overall HrQoL and a higher burden of pelvic symptoms.

## Teaser

The aim of this international, cross-sectional, multicentre, observational study was to understand the short-term health-related quality-of-life (HrQoL) differences between patients undergoing surgical and palliative treatments for locally recurrent rectal cancer (LRRC). The study identifies a number of clinically and statistically significant differences between patients undergoing curative and palliative treatment for LRRC, with patients undergoing curative surgery reporting better overall HrQoL and a higher burden of pelvic symptoms.

## Introduction

The management of locally recurrent rectal cancer (LRRC) has significantly evolved over the last decade, with an expansion in the indications and surgical techniques to achieve cure^1–3^. ‘Higher and wider’ techniques such as high sacrectomy^4–7^ and extended lateral pelvic sidewall excision^8–10^ have expanded the surgical portfolio of available techniques to manage patients with LRRC. Alongside this surgical evolution, there has been an improvement in radiological imaging and navigational technology^11^, and a greater adoption of the use of neoadjuvant treatments, including reirradiation, intraoperative radiotherapy and total neoadjuvant treatment^12^. These combined developments in LRRC have led to improvements in overall survival in patients undergoing surgery, with the international PelvEx group reporting 3-year overall survival of 48 per cent^13^. As the radicality of surgical techniques increases, the associated morbidity, impact on health-related quality of life (HrQoL), and potential for functional deficits also increases.

There has been increasing interest in the impact of surgical techniques on patient-reported outcomes including HrQoL in patients with LRRC, with the Association of Coloproctology of Great Britain and Ireland’s Improving Outcomes in Advanced Colorectal Tumours (IMPACT) initiative^14^, PelvEx and Beyond TME^15^ advocating for outcome reporting in this cohort of patients to include QoL outcomes. Patients with advanced cancer dually value survival and quality of life. HrQoL is particularly important, when survival benefits are potentially limited and must be offset against the risks associated with treatment. Presenting HrQoL data alongside clinical and oncological data is of huge value; promoting shared decision-making and providing a balanced and patient-centric perspective. However, HrQoL is a complex construct and must be appropriately assessed, interpreted, and reported to obtain meaningful results that are generalizable to the wider population and applicable to clinical practice. In LRRC, it is important to understand the HrQoL benefits associated with differing treatment options in the short and long term including the magnitude of change in LRRC HrQoL scores between treatments and over time, as well as the clinical relevance and impact of changes in QoL scores. It is essentially important that HrQoL scores are not interpreted solely on their statistical performance, but on their clinical importance, with the reporting of minimal clinically important differences (MCID). MCID define the smallest change in HrQoL from the patient’s perspective and look beyond statistical significance^16^.

The aim of this study was to conduct a cross-sectional, multicentre, observational cohort to evaluate HrQoL differences between patients undergoing surgical and palliative treatments for LRRC in an international cohort of patients.

## Methods

A multicentre, international, cross-sectional, observational cohort study was undertaken at five centres across the UK and Australia. The study was approved by Yorkshire and The Humber Research Ethics Committee (REC reference: 12/YH/0518) in the UK and by the Sydney Local Health District Ethics Committee in Australia. Patients with LRRC were recruited from prospectively held registries at each participating site; recruitment took place between January 2015 and September 2019. Eligible patients were approached for participation and were appropriately consented for enrolment into the study. Patients were enrolled into the study at varying time points following diagnosis of LRRC, and self-completed validated questionnaires (see below). Clinical and pathological variables were collected, including demographics and socio-economic factors. Clinically relevant categories of patients, including intent of treatment received (curative *versus* palliative), surgical margin status, location of disease (anterior, posterior, central, lateral), and type of palliative treatment received, were compared for their HrQoL outcomes.

### Eligibility criteria

Patient inclusion criteria were age greater than or equal to 18 years old, with an existing resectable LRRC undergoing neoadjuvant treatments or who had undergone surgical treatment with curative or palliative intent for an LRRC within the last 2 years or had undergone non-surgical palliative treatment of LRRC and were able to provide written, informed consent. Exclusion criteria included patients who declined treatment based on individual choice or were considered too frail to pursue either surgical or oncological treatments.

### HrQoL assessment

HrQoL was assessed using two validated questionnaires for use in colorectal cancer; the European Organisation for Research and Treatment of Cancer (EORTC) CR29 and the functional assessment of cancer therapy – colorectal (FACT-C). The FACT-C consists of five subscales: physical well-being, social well-being, emotional well-being, functional well-being, and colorectal cancer subscale^17^. Each item is scored on a 5-point Likert scale, ranging from 0 (not at all) to 4 (very much); higher scores denote good quality of life.

The EORTC QLQ-CR29 consists of 29 items consisting of 4 domains of urinary frequency, blood or mucus in stools, frequency of defaecation, and body image, and 19 single items^18^. Each item is scored on a 4-point Likert scale, ranging from 1 (not at all) to 4 (very much). All scales were linearly converted to a 0–100 scale according to standardized EORTC scoring procedures^19^. For the functioning scales and single items, higher scores indicate better functioning; for the symptom scales and single item, higher scores indicate higher symptom burden.

### Definitions for locally recurrent rectal cancer

LRRC was defined in keeping with the Beyond TME definition of recurrence, progression, or development of new sites of rectal tumour within the pelvis after previous resectional surgery for rectal cancer^15^. Pattern of disease recurrence was classified according to anatomical location: central (anastomotic recurrence after low anterior resection, perineal recurrence following abdominoperineal resection (APR)), anterior (involving urological and/or gynaecological structures), posterior (involving the sacrum and/or coccyx), and lateral (involving pelvic sidewall structures)^20,21^. Options for curative intent included neoadjuvant chemoradiation or chemotherapy and surgical resection. Treatment intent was defined as palliative when patients were offered non-operative management strategies including palliative chemotherapy/radiotherapy and best supportive care due to inoperable local ± distant metastatic disease. Margin status was defined as R0 for a negative microscopic margin, R1 for a positive microscopic margin, and R2 for a positive macroscopic margin.

### Statistical analysis

Clinical and demographic data were summarized descriptively using appropriate frequencies and summary statistics. Summary scores for each scale of the FACT-C and EORTC CR29 were calculated for each patient. The completeness of HrQoL data was analysed for FACT-C and EORTC CR29 to identify missing data at item and scale level. The criteria for acceptable levels of missing data were less than 10 per cent for items and less than 50 per cent for computable total scale scores. Handling of missing data for each scale was conducted using half-mean imputation. If half or more of the items within the scale were complete, the missing items within the scale could be imputed with the mean of the remaining items^22^. To assess the magnitude of the observed differences, Cohen effect sizes (ESs) were calculated. ESs of 0.2, 0.5, and 0.8 were considered small, moderate, and large respectively. MCID for FACT-C were 2–3 points for the colorectal cancer subscale, 4–6 points for the treatment outcome index (the sum of the colorectal cancer subscale, physical well-being, and functional well-being subscales) and 5–8 points for the total FACT-C score^23^. For the EORTC QLQ-C30, a 5–10 mean score difference was regarded as small, but subjectively significant or clinically meaningful, a 10–20-point change was regarded as moderate, and a greater than 20-point change was regarded as large^24–26^. More recently, the EORTC group has determined that a 5–10 mean score difference in advanced colorectal cancer was clinically relevant using scores from the EORTC QLQ-C30^27^. HrQoL data at a domain or scale level, including mean differences, were analysed in clinically relevant categories using the independent *t* test to examine differences in mean scores for two groups and the one-way analysis of variance (ANOVA) for more than two groups.

## Results

### Patient and clinical characteristics

Out of 305 eligible patients, a total of 95 patients participated in this study with 66 (69.5 per cent) male participants with a median age of 66 (interquartile range (i.q.r.) 57–71) years. Seventy-four (77.8 per cent) patients were treated surgically with curative intent and 21 (22.1 per cent) were treated with palliative intent. All patients undergoing surgical treatment did so with curative intent, there were no patients treated surgically with palliative intent enrolled into this study. Median time from treatment of primary rectal cancer to diagnosis of LRRC was 24 (i.q.r. 2–43) months. Primary rectal cancer tumour treatment details are provided in the *Supplementary material* (*Table S1*). There were no differences in gender, mean age, ethnicity, or educational status between the two groups (*Table 1*). There were some observed differences between the two groups with regards to employment status, with a higher proportion of patients in the palliative group on sick leave when compared with the surgical group (28.5 per cent versus 2.7 per cent, *P* = 0.001). The anatomical pattern of pelvic disease was similar between the two groups (*P* = 0.176); however, patients treated palliatively had a higher proportion of metastatic disease compared with the surgical group (42.9 per cent versus 2.7 per cent, *P* < 0.001).

**Table 1:** Patient demographic and clinical characteristics

Variable	Surgical treatment	Palliative treatment	*P*
**Gender**			
Male	51 (68.9)	15 (71.4)	0.823
Female	23 (31.1)	6 (28.6)	
**Age (years), mean(s.d.)**	65.3(9.8)	63.2(11.7)	0.430
**Ethnicity**			
White	31 (41.8)	19 (90.4)	0.175
Black	3 (4.0)	0 (0.0)	
Asian	0 (0.0)	1 (4.7)	
Unknown	40 (54.5)	1 (4.7)	
**Marital status**			
Married	58 (78.3)	17 (80.9)	0.078
Living common law	1 (1.3)	1 (4.7)	
Widowed	0 (0.0)	2 (9.5)	
Separated	2 (2.7)	0 (0.0)	
Divorced	3 (4.0)	0 (0.0)	
Single	3 (4.0)	0 (0.0)	
Unknown	7 (9.4)	1 (4.7)	
**Educational status**			
Secondary school	29 (39.1)	7 (33.3)	0.216
College	16 (21.6)	6 (28.5)	
University	13 (17.5)	7 (33.3)	
Other	8 (10.8)	0 (0.0)	
Unknown	8 (10.8)	1 (4.7)	
**Employment status**			
Self-employed	7 (9.4)	2 (9.5)	0.012
Homemaker	1 (1.3)	0 (0.0)	
Full-time employment	7 (9.4)	0 (0.0)	
Part-time employment	4 (5.4)	2 (9.5)	
Unemployed	1 (1.3)	0 (0.0)	
Sick leave	2 (2.7)	6 (28.5)	
Retired	43 (58.1)	9 (42.8)	
Unknown	9 (12.1)	2 (9.5)	
**Mode of detection**			
Symptomatic	22 (29.7)	5 (23.8)	0.411
Screening	44 (59.4)	16 (76.2)	
Unknown	8 (10.8)	0 (0.0)	
**Pattern of recurrence**			
Anterior	11 (14.8)	1 (4.8)	0.176
Central	27 (36.4)	6 (28.6)	
Posterior	17 (22.9)	10 (47.6)	
Lateral	9 (12.1)	4 (19.0)	
Unknown	10 (13.5)	0 (0.0)	
**Presence of metastatic disease**			
Yes	2 (2.7)	9 (42.9)	<0.001
No	72 (97.3)	12 (57.1)	

Values are *n* (%) unless otherwise indicated.

Of the 21 patients who were treated with palliative intent, 16 (76.2 per cent) patients underwent chemotherapy and 5 (23.8 per cent) patients underwent chemoradiation. Of the patients treated surgically, total pelvic exenteration was the most performed operation (28 patients, 37.8 per cent), followed by posterior pelvic exenteration (10 patients, 13.5 per cent). Ten (13.5 per cent) patients underwent a sacrectomy and seven (9.4 per cent) patients underwent an extended pelvic sidewall excision. Surgical treatment details are highlighted in *Table S2*.

### HrQoL assessment

The median time between date of diagnosis of LRRC and completion of HrQoL assessments was 4 (i.q.r. 2–7) months. The median time between date of surgery and completion of HrQoL assessments was 3 (i.q.r. 1–5) months.

### FACT-C scores

**Fig. 1:**
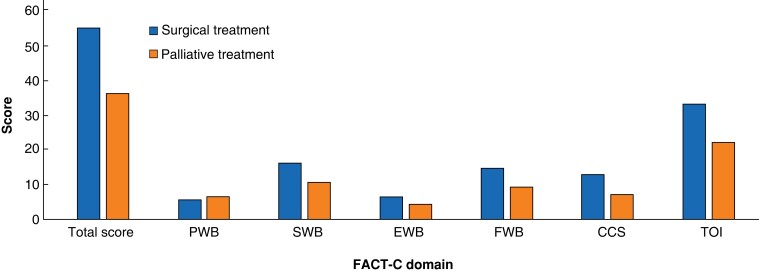
Functional assessment of cancer therapy – colorectal scores in surgical and palliative treatment groups PWB, physical well-being; SWB, social well-being; EWB, emotional well-being; FWB, functional well-being; CCS, colorectal cancer subscale; TOI, treatment outcome index.

Overall HrQoL as measured by FACT-C was better in patients undergoing curative treatment for LRRC compared with palliative treatment, from a statistical and clinical perspective, with mean FACT-C scores of 55.1 and 36.5 (*P* < 0.001) respectively, a moderate ES of 0.6, and a demonstrable MCID, with a mean difference of 18.5 between treatment scores (*Table 2* and *Fig. 1*). FACT-C demonstrated statistically significant higher HrQoL scores in patients undergoing surgical treatment within the domains of social, emotional, and functional well-being. The FACT-C colorectal cancer subscale demonstrated a clinically relevant higher QoL score in surgical patients compared with palliative patients (12.9 and 7.2) respectively, with an MCID of 5.78 and a large ES of 0.8, (*P* = 0.090). Clinically relevant differences in scores were observed in the colorectal cancer subscale, the treatment outcome index and the overall scores, suggesting better HrQoL in patients undergoing surgery.

**Table 2:** FACT-C health-related quality-of-life scores

FACT-C quality-of-life domains	Curative intent scores, mean(s.d.)	Palliative intent scores, mean(s.d.)	Mean difference	ES	*P*	MCID
Physical well-being	5.68(4.97)	6.52(7.39)	−0.83	0.1	0.002	N/A
Social well-being	16.2(8.04)	10.7(9.63)	5.49	0.6	0.011	N/A
Emotional well-being	6.49(3.93)	4.52(4.98)	1.96	0.4	0.017	N/A
Functional well-being	14.8(7.55)	9.3(10.51)	5.59	0.6	0.004	N/A
Colorectal cancer subscale	12.9(6.88)	7.2(7.24)	5.78	0.8	0.009	Yes
Treatment outcome index	33.3(15.64)	22.3(22.37)	11.0	0.6	0.018	Yes
Overall FACT-C score	55.1(26.29)	36.5(36.64)	18.5	0.6	0.018	Yes

ES, effect size; MCID, minimal clinically important differences; N/A, non applicable.

### EORTC CR29 scores

**Fig. 2:**
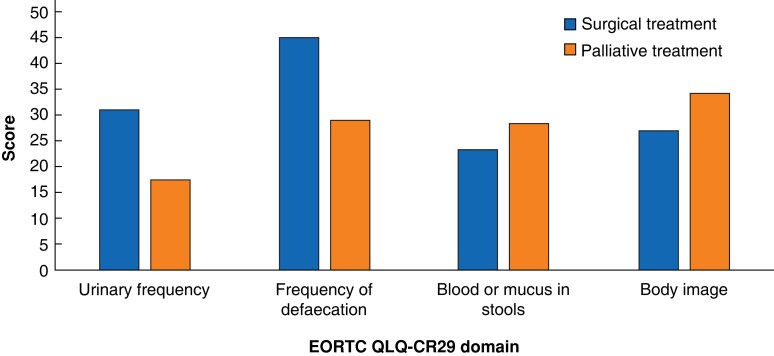
EORTC QLQ-CR29 domain scores in surgical and palliative treatment groups

**Fig. 3:**
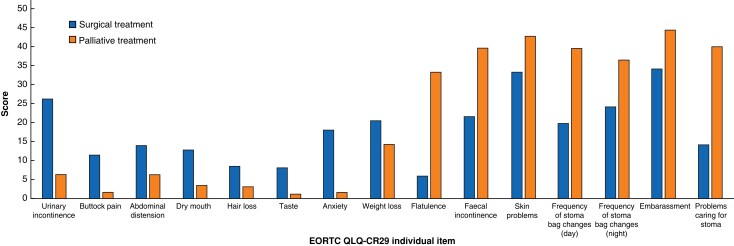
EORTC QLQ-CR29 individual item scores in surgical and palliative treatment groups

The EORTC CR29 domains of urinary frequency and frequency of defaecation demonstrated clinically and statistically significant differences between surgical and palliative patients, with a higher burden of symptoms and worse HrQoL scores in the surgical cohort (*Table 3* and *Fig. 2*). The blood or mucus scale and body image scale did not demonstrate any statistically significant differences between the two groups, but did demonstrate clinically relevant differences, with worse HrQoL scores observed in the palliative setting. The calculated ES for differences between the two treatment groups across all EORTC CR29 domains was small. Statistically and clinically significant differences were detected between the two groups in the EORTC CR29 single items of urinary incontinence, dry mouth, and anxiety, with a higher burden of symptoms and worse HrQoL in the surgical cohort (*Table 4* and *Fig. 3*). MCID with small to moderate ESs were observed in the EORTC CR29 items measuring buttock pain, abdominal distension, hair loss, taste, and weight; with higher HrQoL scores in the surgical cohort. Conversely, the EORTC CR29 items assessing flatulence, incontinence, frequency of bag changes during the day and night, embarrassment and problems identified MCID in HrQoL scores with moderate to large ESs in the palliative cohort.

**Table 3:** EORTC CR29 domain scores

EORTC CR29 quality-of-life domains	Curative intent scores, mean(s.d.)	Palliative intent scores, mean(s.d.)	Mean difference	ES	*P*	MCID
Urinary frequency	31.2(47.4)	17.5(39.8)	11.65	0.3	<0.001	Yes
Frequency of defaecation	45.4(32.3)	29.1(36.9)	10.62	0.4	<0.001	Yes
Blood or mucus in stools	23.3(16.5)	28.5(19.8)	7.69	0.3	0.201	Yes
Body image	27.2(21.1)	34.3(28.3)	7.17	0.3	0.179	Yes

Higher scores indicate higher symptom burden and worse health-related quality of life. ES, effect size; MCID, minimal clinically important differences.

**Table 4:** EORTC CR29 single-item scores

EORTC CR29 single items	Curative intent scores, mean(s.d.)	Palliative intent scores, mean(s.d.)	Mean difference	ES	*P*	MCID
Urinary incontinence	26.2(18.6)	6.3(8.8)	19.8	0.5	0.033	Yes
Dysuria	3.2(6.7)	4.8(3.2)	0.93	0.1	0.343	No
Abdominal pain	8.1(7.1)	9.5(6.5)	1.45	0.1	0.835	No
Buttock pain	11.4(8.6)	1.7(1.3)	9.62	0.3	0.107	Yes
Abdominal distension	14.0(12.1)	6.3(3.2)	7.66	0.2	0.407	Yes
Dry mouth	12.8(4.5)	3.5(1.6)	8.09	0.8	<0.010	Yes
Hair loss	8.6(6.8)	3.2(2.9)	7.31	0.1	0.113	Yes
Taste	8.1(7.6)	1.2(0.5)	7.09	0.2	0.335	Yes
Anxiety	18.1(12.1)	1.6(1.0)	17.68	0.5	0.011	Yes
Weight loss	20.5(22.7)	14.3(8.8)	6.19	0.1	0.523	Yes
Flatulence	5.9(5.3)	33.3(23.8)	27.4	0.7	0.011	Yes
Faecal incontinence	21.6(22.9)	39.6(19.0)	18.1	0.5	0.095	Yes
Skin problems	33.3(28.7)	42.8(26.9)	9.52	0.3	0.238	Yes
Frequency of stoma bag changes (day)	19.8(12.6)	39.6(22.3)	19.8	0.5	0.051	Yes
Frequency of stoma bag changes (night)	24.1(26.4)	36.5(28.5)	12.4	0.3	0.266	Yes
Embarrassment	34.2(32.2)	44.4(32.7)	10.21	0.4	0.638	Yes
Problems caring for stoma	14.2(8.5)	40.0(35.9)	25.18	0.5	0.075	Yes

ES, effect size; MCID, minimal clinically important differences.

### Patient and clinical variables

Higher overall FACT-C scores denoting better HrQoL were observed in patients who underwent an R0 resection compared with an R1 resection, with scores of 56.5 and 49.0 respectively (*P* = 0.051; see *Table 5*), an ES of 0.6, and a clinically significant MCID of 7.5 between treatment scores. FACT-C social well-being scores were higher in patients who attended university compared with patients who had attended college, with scores of 16.8 and 11.45 respectively (*P* = 0.031); ES 0.6. EORTC CR29 scores identified worse body image in patients with posterior and central disease compared with anterior and lateral disease from a statistical and clinical perspective (*P* = 0.021) (*Table 6*). Patients undergoing palliative chemoradiation reported worse HrQoL scores with a higher symptom burden on the frequency of defaecation scale compared with palliative chemotherapy, with EORTC CR29 scores of 50.0 and 35.9 respectively (*P* = 0.041) (see *Table 6*).

**Table 5:** FACT-C health-related quality-of-life scores in clinical categories

Clinical variable	Physical well-being scores, mean(s.d.)	Social well-being scores, mean(s.d.)	Emotional well-being scores, mean(s.d.)	Functional well-being scores, mean(s.d.)	Colorectal cancer subscale scores, mean(s.d.)	Treatment outcome index scores, mean(s.d.)	Overall scores, mean(s.d.)
**Pattern of disease**							
Anterior	7.0(3.6)	12.7(6.9)	4.6(3.8)	11.0(6.5)	9.1(6.0)	26.9(13.1)	43.4(23.5)
Central	4.9(3.8)	18.4(8.4)	6.5(4.5)	17.8(8.7)	13.0(7.4)	35.0(18.2)	35.0(30.6)
Lateral	6.4(5.9)	14.4(8.7)	5.6(4.1)	13.1(9.0)	11.1(6.9)	30.5(19.3)	38.4(31.3)
Posterior	4.75(3.1)	13.8(9.6)	7.0(5.2)	13.5(11.6)	11.8(8.0)	29.1(22.9)	39.0(36.8)
*P*	0.537	0.413	0.301	0.295	0.487	0.569	0.698
**Palliative treatment**							
Chemotherapy	5.7(6.8)	10.25(9.4)	4.7(4.7)	10.2(12.1)	6.5(7.3)	22.5(15.6)	37.5(27.1)
Chemoradiation	5.9(6.6)	10.25(11.8)	4.0(5.8)	8.8(10.8)	6.8(7.5)	20.2(16.8)	33.0(23.4)
*P*	0.463	0.635	0.316	0.913	0.562	0.461	0.451
**Preoperative treatment**							
None	6.1(5.7)	18.2(8.4)	6.0(3.7)	15.5(9.4)	13.5(7.3)	34.8(20.5)	59(33.2)
Chemoradiation	6.2(5.6)	16.3(7.1)	7.0(3.6)	16.1(8.2)	14.0(7.5)	36.4(21.1)	59(30.7)
*P*	0.973	0.595	0.584	0.932	0.886	0.928	0.995
**Margin status**							
R0	6.1(5.4)	16.7(8.3)	6.7(3.8)	15.0(8.7)	13.5(8.7)	34.4(18.2)	56.5(28.3)
R1	5.4(4.3)	14.2(8.1)	5.7(4.2)	13.9(9.1)	10.6(9.1)	29.7(19.6)	49.0(31.3)
*P*	0.261	0.179	0.834	0.293	0.493	0.051	0.051
**Postoperative treatments**							
Chemotherapy	4.0(5.3)	13.6(7.4)	5.5(3.6)	11.9(7.3)	8.8(6.6)	31.3(15.1)	41.3(24.6)
None	6.7(5.3)	16.2(8.7)	6.2(4.1)	15.0(7.5)	13.2(7.0)	23.7(16.0)	57.1(28.4)
*P*	0.201	0.041	0.456	0.387	0.379	0.110	0.060

**Table 6:** EORTC CR29 health-related quality-of-life scores in clinical categories

Clinical variable	Urinary frequency scores, mean(s.d.)	Frequency of defaecation scores, mean(s.d.)	Blood or mucus in stools scores, mean(s.d.)	Body image scores, mean(s.d.)
**Pattern of disease**				
Anterior	29.5(8.8)	19.4(8.3)	1.9(2.3)	19.1(13.4)
Central	7.2(6.2)	5.2(6.2)	1.5(2.6)	31.0(20.1)
Lateral	15.3(8.5)	6.2(3.1)	3.1(2.8)	25.2(17.2)
Posterior	20.8(8.1)	8.3(7.4)	5.0(4.3)	40.5(22.5)
*P*	0.643	0.572	0.767	0.021
**Palliative treatment**				
Chemotherapy	20.0(15.8)	35.9(26.4)	4.6(3.8)	34.0(19.3)
Chemoradiation	12.5(10.3)	50.0(32.4)	12.5(6.5)	27.7(12.5)
*P*	0.720	0.041	0.576	0.979
**Preoperative treatment**				
None	33.3(25.8)	20.0(17.6)	7.9(4.3)	30.1(25.8)
Chemoradiation	38.2(2.1)	27.5(22.1)	5.3(2.9)	25.1(20.3)
*P*	0.881	0.420	0.681	0.524
**Margin status**				
R0	35.0(24.3)	21.6(17.7)	4.7(2.3)	30.4(26.5)
R1	14.7(12.2)	23.4(19.8)	2.9(1.2)	29.4(24.2)
*P*	0.293	0.445	0.956	0.071
**Postoperative treatments**				
Chemotherapy	5.5(3.3)	7.5(5.1)	1.3(1.1)	27.7(24.3)
None	32.5(25.7)	23.1(19.8)	1.6(1.3)	30.8(26.8)
*P*	0.401	0.865	0.142	0.091

## Discussion

This study highlights the complexity of the treatment impact on HrQoL in an international cohort of patients with LRRC, identifying statistical and clinically significant differences in patients undergoing surgery compared with palliative treatments. This study demonstrates better overall short-term HrQoL from a clinical and statistical standpoint, as measured by FACT-C, in patients with LRRC undergoing surgery with a higher symptom burden and worse HrQoL scores in local pelvic symptoms, as measured by EORTC CR29, specifically with regards to urinary frequency and incontinence, and frequency of defaecation. Clinical factors associated with improved HrQoL scores across a range of domains included negative margin status, anterior pattern of disease, and the use of palliative chemotherapy.

The study presents a comprehensive overview of colorectal-specific HrQoL in the immediate interval following diagnosis for patients with LRRC. This may be more pertinent than generic HrQoL as treatments are initiated and new symptoms develop due to a combination of the disease and treatment effects. Assessing HrQoL provides a patient-centric perspective on understanding treatment effects; combining this assessment with MCID in the interpretation of these scores allows for a truly patient-centric perspective. Employing this approach identified several important clinical differences in HrQoL between patients undergoing surgery compared with palliative treatments. It is interesting to note that ESs were small to moderate in the items demonstrating worse HrQoL outcomes for the surgical cohort, compared with the moderate to large ESs in the palliative cohort. This suggests that the impact of worse HrQoL scores is more pronounced in patients undergoing palliative treatment. Understanding the differences in HrQoL issues based on treatment strategy has important implications in understanding the early clinical course following diagnosis and treatment, surgical or palliative, and should inform decision-making and informed consent in this complex cohort. It should also focus the development of targeted clinical strategies to maintain and improve HrQoL during this time frame. Shared decision-making with patients should take into account a number of key findings from our study. This includes the deterioration and/or development of new pelvic symptoms in patients undergoing surgery compared with worsening overall colorectal symptoms in patients undergoing palliative treatments. This is key to improving patient understanding of post-treatment symptom trajectory and managing patient expectations appropriately pre-treatment. The lower social, emotional, and functional well-being scores demonstrated in the palliative cohort must also be acknowledged with appropriate supportive strategies identified to help support patients and improve QoL.

A negative margin status (R0) was associated with better overall HrQoL compared with a positive margin, as measured by the FACT-C questionnaire. Margin status has been consistently shown to be a prognostic marker in LRRC for overall survival^13,28^. Associating improved HrQoL in patients with an R0 margin status has the ability to enhance the improvement in survival alone in this cohort of patients. ‘Quality of survival’ extends traditional survival metrics to include HrQoL, survival, treatment-related effects, and economic impact^29^, and may be of greater value when considering treatment options in complex clinical scenarios such as LRRC.

Relevant MCID were not available for all domains of the FACT-C; social, emotional, and functional well-being. In these domains we report significantly better HrQoL in those undergoing surgery compared with palliative treatments; however, we were unable to quantify the clinical impact of this. Parameters for MCID for FACT-C and EORTC CR29 were taken from scores and clinical anchors relevant to patients with advanced primary colorectal cancer, and, therefore, must be interpreted with caution, as they may not be applicable to patients with LRRC. Furthermore, the MCID for EORTC CR29 were extrapolated from scores obtained from the EORTC QLQ-C30^27^. To mitigate for this, we also calculated the ES of the observed differences to identify the magnitude of HrQoL differences to provide clinical context. One of the consistent methodological drawbacks in assessing HrQoL in LRRC is the lack of validated, disease-specific instruments available in this cohort of patients^30,31^. Previous works have identified several wide-ranging HrQoL issues and symptomatology affecting patients with LRRC^30,32^; many of which are not captured in outcome measures designed for use in primary colorectal cancer. This may account for the fact that a clinically and statistically significant difference in the FACT-C colorectal cancer subscale between surgically treated and palliatively treated patients was not documented, despite finding several differences within the EORTC CR29 domains and items. The measurement properties of the FACT-C and EORTC CR29 differ slightly, with the FACT-C colorectal cancer subscale assessing gastrointestinal issues and the EORTC CR29 providing broader assessment of pelvic issues^33^. The lack of discrimination by the FACT-C reflects its inability to capture the clinical complexity of pelvic disease in LRRC, which often involves a number of compartments within the pelvis and manifests primarily with pelvic symptoms. In view of the relatively small sample size, particularly with regards to the palliative group, coupled with multiple statistical testing, these results should be interpreted with caution.

Significant strides have been made over the last decade to understand the impact of LRRC on HrQoL^30^. The adverse impact of LRRC on all domains of HrQoL has been widely acknowledged^34^. Previous works have reported a return to baseline HrQoL within a 6–12-month interval depending on the complexity of surgery using the FACT-C questionnaire^10,35^, with maintenance of HrQoL scores in long-term survivors over a 5-year interval. The lack of benefit to improving QoL in the palliative surgical setting has also been demonstrated using FACT-C^36,37^. HrQoL in LRRC is a complex construct but with prognostic implications; with baseline scores predicting postoperative HrQoL outcomes^38–40^. Although, these works demonstrate the clear impact of surgery in improving HrQoL in LRRC, they are limited to single-centre cohort studies and case series, with a variable number of patients utilizing several differing outcome measures to assess HrQoL^30,31^. The present work contributes to the growing body of evidence on the impact of LRRC and its treatment of HrQoL reporting short-term HrQoL benefits in surgery at a domain level from a clinical perspective. This is the first multicentre, international, cross-sectional, observational cohort study comparing short-term HrQoL outcomes between surgical and palliatively treated patients. Several clinically and statistically significant differential outcomes in HrQoL scores between the two cohorts were highlighted across a range of items and domains. The multicentre nature of this work begins to explore the potential generalizability and applicability of our HrQoL results to other exenterative centres internationally. Future works will focus on assessing longitudinal, disease-specific HrQoL in patients with LRRC to identify the specific and long-term treatment effects, considering the clinical significance of changes in HrQoL scores.

## Supplementary Material

zrac168_Supplementary_Data

## Data Availability

Data may be available on request from the authors subject to privacy/ethical restrictions.
