# Supplementary material for: Short-term outcomes of health-related quality of life in patients with locally recurrent rectal cancer: multicentre, international, cross-sectional cohort study
Source: BJS Open. 2023 Feb 14;7(1):zrac168. doi: 10.1093/bjsopen/zrac168 (PMC9927560; doi:10.1093/bjsopen/zrac168)
Supplement: zrac168_Supplementary_Data [file zrac168_supplementary_data.docx]

**Short term outcomes of Health-Related Quality of Life in Patients with Locally Recurrent Rectal Cancer: multi-centre, international, cross-sectional cohort study**

Miss Deena P. Harji^1, 2^

Miss Niamh McKigney^1^

Professor Cherry Koh^,3,4,5,6^

Professor Michael J. Solomon^,3,4,5,6^

Mr Ben Griffiths^2^

Mr Martyn Evans^7^

Professor Alexander Heriot^8^

Professor Peter M. Sagar^9^

Professor Galina Velikova^10,11^

Professor Julia M. Brown^1^

1. *Clinical Trials Research Unit, Leeds Institute of Clinical Trials Research, University of Leeds, Leeds, UK.*
2. *Manchester University NHS Foundation Trust, Manchester, UK*
3. *Surgical Outcomes Research Centre (SOuRCe), Royal Prince Alfred Hospital, Sydney, NSW, Australia.*
4. *Faculty of Medicine and Health, Central Clinical School, The University of Sydney, Sydney, NSW, Australia.*
5. *RPA Institute of Academic Surgery, Royal Prince Alfred Hospital, Sydney, NSW, Australia.*
6. *Department of Colorectal Surgery, Royal Prince Alfred Hospital, Sydney, NSW, Australia.*
7. *Heol Maes Eglwys, Morriston, Swansea, Wales.*
8. *Sir Peter MacCallum Department of Oncology, University of Melbourne, Melbourne, Victoria, Australia.*
9. *The John Goligher Department of Colorectal Surgery, St. James’s University Hospital, Leeds, LS7 9TF, UK*
10. *Leeds Institute of Medical Research, University of Leeds, Leeds, UK*
11. *St James's Institute of Oncology, St James's University Hospital, Leeds, UK.*

**Corresponding author:** Miss Deena Harji**,** Manchester University NHS Foundation Trust, Manchester **Twitter:** @DeenaHarji

**Supplementary Materials – Index**

| **Supplementary Figures and Tables** |  |
| --- | --- |
| Table S1: Primary Rectal Cancer Tumour Characteristics | *pag. 3* |
| Table S2: Surgical Treatment Characteristics | *pag. 4* |
|  |  |

**Table S1: Primary Rectal Cancer Tumour Characteristics**

| **Variable** | **Curative Treatment** | **Palliative Treatment** | **P Value** |
| --- | --- | --- | --- |
| **Neoadjuvant Treatment**  None  Short course radiotherapy  Chemoradiation  Chemotherapy  Unknown | 42 (56.7)  1 (1.3)  22 (29.7)  6 (8.1)  3 (4.1) | 7 (33.3)  3 (14.3)  10 (47.6)  1 (4.8)  0 (0.0) | 0.005 |
| **Primary Operation**  Anterior Resection  Abdominoperineal excision rectum  Composite Abdominosacral Resection  Hartmaan’s Procedure  Local excision  Panproctocolectomy  Pelvic Exenteration  Unknown | 45 (60.8)  13 (17.5)  0 (0.0)  3 (4.1)  3 (4.1)  1 (1.4)  0 (0.0)  9 (12.1) | 12 (57.1)  2 (9.5)  2 (9.5)  3 (14.3)  1 (4.8)  0 (0.0)  1 (4.8)  0 (0.0) | 0.01 |
| **TNM – T Stage**  T1  T2  T3  T4  Unknown | 1 (1.3)  9 (12.1)  33 (44.5)  11 (14.8)  20 (27.0) | 3 (14.3)  3 (14.3)  10 (47.6)  5 (23.8)  0 (0.0) | 0.02 |
| **TNM – N Stage**  N0  N1  N2  Unknown | 22 (29.7)  26 (35.1)  6 (8.1)  20 (27.0) | 14 (66.6)  7 (33.3)  0 (0.0)  0 (0.0) | 0.02 |
| **Overall number staging**  Stage 1  Stage 2  Stage 3  Stage 4  Unknown | 7 (9.5)  15 (20.3)  32 (43.2)  0 (0.0)  20 (27.0) | 5 (28.3)  9 (42.9)  7 (33.3)  0 (0.0)  0 (0.0) | 0.06 |
| **Margin Status**  R0  R1  Unknown | 46 (62.1)  20 (27.0)  8 (10.8) | 19 (90.5)  2 (9.5)  (0.0) | 0.05 |
| **Adjuvant Treatment**  None  Chemotherapy  Chemoradiation  Unknown | 15 (20.2)  20 (27.0)  15 (20.2)  24 (32.4) | 9 (42.9)  11 (52.4)  1 (4.8)  0 (0.0) | <0.001 |

**Table S2: Surgical Treatment Characteristics**

| **Variable** | Number (%) |
| --- | --- |
| **Operation** |  |
| Composite Abdominosacral Resection | 6 (8.1) |
| Anoproctecomy | 1 (1.3) |
| Abdominoperineal resection | 7 (9.4) |
| Excision of recurrent rectal cancer with en bloc vaginectomy | 1 (1.3) |
| Resection of neorectum with segment 6 metastatectomy | 1 (1.3) |
| Hartman’s | 3 (4.0) |
| Laparotomy and bypass of recurrent rectal cancer | 1 (1.3) |
| Total Pelvic Exenteration | 28 (37.8) |
| Posterior Pelvic Exenteration | 8 (10.8) |
| Protectomy with en bloc vaginectomy | 1(1.3) |
| Resection neorectum | 1(1.3) |
| Pelvic sidewall resection | 7 (9.4) |
| Resection of recurrent rectal cancer | 2 (5.4) |
| Resection of recurrent rectal cancer with en bloc small bowel | 1(1.3) |
| Resection of recurrent rectal cancer with en bloc hysterectomy and bilateral oophorectomy | 1 (1.3) |
| Resection neorectum | 1 (1.3) |
| Resection of recurrent rectal cancer with en bloc iliacus and psoas muscle | 1 (1.3) |
| Trans-sacral resection of mass | 1 (1.3) |
| Trans-perineal resection of mass | 1 (1.3) |
| Ultralow anterior resection with en bloc resection of right obturator node | 1 (1.3) |
| **Pre-operative Treatment** |  |
| None | 24 (32.4) |
| Chemoradiation | 37 (50.0) |
| Unknown | 13 (17.5) |
| **Margin Status** |  |
| R0 | 44 (59.5) |
| R1 | 17 (23.0) |
| Unknown | 13 (17.6) |
| **Post-Operative Treatments** |  |
| Chemotherapy | 10 (13.5) |
| None | 46 (62.2) |
| Unknown | 18 (24.3) |
